# Supplementary material for: Iterative improvement in the automatic modular design of robot swarms
Source: PeerJ Comput Sci. 2020 Dec 7;6:e322. doi: 10.7717/peerj-cs.322 (PMC7924708; doi:10.7717/peerj-cs.322)
Supplement: Supplemental Information 3 [file peerj-cs-06-322-s003.zip › argos3/doc/api/standalone/a00624_source.html]

ARGoS: plugins/simulator/physics\_engines/pointmass3d/pointmass3d\_engine.h Source File


- Main Page
- Related Pages
- Namespaces
- Classes
- Files

- File List
- File Members

# plugins/simulator/physics\_engines/pointmass3d/pointmass3d\_engine.h

Go to the documentation of this file.

```
00001 
00007 #ifndef POINTMASS3D_ENGINE_H
00008 #define POINTMASS3D_ENGINE_H
00009 
00010 namespace argos {
00011    class CPointMass3DEngine;
00012    class CPointMass3DModel;
00013    class CEmbodiedEntity;
00014 }
00015 
00016 #include <argos3/core/utility/math/ray2.h>
00017 #include <argos3/core/simulator/entity/controllable_entity.h>
00018 #include <argos3/core/simulator/physics_engine/physics_engine.h>
00019 
00020 namespace argos {
00021 
00022    class CPointMass3DEngine : public CPhysicsEngine {
00023 
00024    public:
00025 
00026       CPointMass3DEngine();
00027       virtual ~CPointMass3DEngine();
00028 
00029       virtual void Init(TConfigurationNode& t_tree);
00030       virtual void Reset();
00031       virtual void Destroy();
00032 
00033       virtual void Update();
00034 
00035       virtual size_t GetNumPhysicsModels();
00036       virtual bool AddEntity(CEntity& c_entity);
00037       virtual bool RemoveEntity(CEntity& c_entity);
00038 
00039       virtual bool IsPointContained(const CVector3& c_point);
00040 
00041       virtual bool IsEntityTransferNeeded() const;
00042 
00043       virtual void TransferEntities();
00044 
00045       virtual void CheckIntersectionWithRay(TEmbodiedEntityIntersectionData& t_data,
00046                                             const CRay3& c_ray) const;
00047 
00048       void AddPhysicsModel(const std::string& str_id,
00049                            CPointMass3DModel& c_model);
00050       void RemovePhysicsModel(const std::string& str_id);
00051 
00052       std::map<std::string, CPointMass3DModel*>& GetPhysicsModels() {
00053          return m_tPhysicsModels;
00054       }
00055 
00056       const std::map<std::string, CPointMass3DModel*>& GetPhysicsModels() const {
00057          return m_tPhysicsModels;
00058       }
00059 
00060       inline Real GetGravity() const {
00061          return m_fGravity;
00062       }
00063 
00064    private:
00065 
00066       CControllableEntity::TMap m_tControllableEntities;
00067       std::map<std::string, CPointMass3DModel*> m_tPhysicsModels;
00068       Real m_fGravity;
00069 
00070    };
00071 
00072    /****************************************/
00073    /****************************************/
00074 
00075    template <typename ACTION>
00076    class CPointMass3DOperation : public CEntityOperation<ACTION, CPointMass3DEngine, SOperationOutcome> {
00077    public:
00078       virtual ~CPointMass3DOperation() {}
00079    };
00080 
00081    class CPointMass3DOperationAddEntity : public CPointMass3DOperation<CPointMass3DOperationAddEntity> {
00082    public:
00083       virtual ~CPointMass3DOperationAddEntity() {}
00084    };
00085 
00086    class CPointMass3DOperationRemoveEntity : public CPointMass3DOperation<CPointMass3DOperationRemoveEntity> {
00087    public:
00088       virtual ~CPointMass3DOperationRemoveEntity() {}
00089    };
00090 
00091 #define REGISTER_POINTMASS3D_OPERATION(ACTION, OPERATION, ENTITY)       \
00092    REGISTER_ENTITY_OPERATION(ACTION, CPointMass3DEngine, OPERATION, SOperationOutcome, ENTITY);
00093 
00094 #define REGISTER_STANDARD_POINTMASS3D_OPERATION_ADD_ENTITY(SPACE_ENTITY, PM3D_MODEL) \
00095    class CPointMass3DOperationAdd ## SPACE_ENTITY : public CPointMass3DOperationAddEntity { \
00096    public:                                                              \
00097    CPointMass3DOperationAdd ## SPACE_ENTITY() {}                        \
00098    virtual ~CPointMass3DOperationAdd ## SPACE_ENTITY() {}               \
00099    SOperationOutcome ApplyTo(CPointMass3DEngine& c_engine,              \
00100                              SPACE_ENTITY& c_entity) {                  \
00101       PM3D_MODEL* pcPhysModel = new PM3D_MODEL(c_engine,                \
00102                                                c_entity);               \
00103       c_engine.AddPhysicsModel(c_entity.GetId(),                        \
00104                                *pcPhysModel);                           \
00105       c_entity.                                                         \
00106          GetComponent<CEmbodiedEntity>("body").                         \
00107          AddPhysicsModel(c_engine.GetId(), *pcPhysModel);               \
00108       return SOperationOutcome(true);                                   \
00109    }                                                                    \
00110    };                                                                   \
00111    REGISTER_POINTMASS3D_OPERATION(CPointMass3DOperationAddEntity,       \
00112                                   CPointMass3DOperationAdd ## SPACE_ENTITY, \
00113                                   SPACE_ENTITY);
00114 
00115 #define REGISTER_STANDARD_POINTMASS3D_OPERATION_REMOVE_ENTITY(SPACE_ENTITY) \
00116    class CPointMass3DOperationRemove ## SPACE_ENTITY : public CPointMass3DOperationRemoveEntity { \
00117    public:                                                              \
00118    CPointMass3DOperationRemove ## SPACE_ENTITY() {}                     \
00119    virtual ~CPointMass3DOperationRemove ## SPACE_ENTITY() {}            \
00120    SOperationOutcome ApplyTo(CPointMass3DEngine& c_engine,              \
00121                 SPACE_ENTITY& c_entity) {                               \
00122       c_engine.RemovePhysicsModel(c_entity.GetId());                    \
00123       c_entity.                                                         \
00124          GetComponent<CEmbodiedEntity>("body").                         \
00125          RemovePhysicsModel(c_engine.GetId());                          \
00126       return SOperationOutcome(true);                                   \
00127    }                                                                    \
00128    };                                                                   \
00129    REGISTER_POINTMASS3D_OPERATION(CPointMass3DOperationRemoveEntity,    \
00130                                   CPointMass3DOperationRemove ## SPACE_ENTITY, \
00131                                   SPACE_ENTITY);
00132 
00133 #define REGISTER_STANDARD_POINTMASS3D_OPERATIONS_ON_ENTITY(SPACE_ENTITY, PM3D_ENTITY) \
00134    REGISTER_STANDARD_POINTMASS3D_OPERATION_ADD_ENTITY(SPACE_ENTITY, PM3D_ENTITY) \
00135    REGISTER_STANDARD_POINTMASS3D_OPERATION_REMOVE_ENTITY(SPACE_ENTITY)
00136 
00137    /****************************************/
00138    /****************************************/
00139 
00140 }
00141 
00142 #endif
```

---

Generated on 10 Jul 2018 for ARGoS by 
 1.6.1
